# Bis(1,10-phenanthroline-κ^2^ *N*,*N*′)(sulfato-κ^2^ *O*,*O*′)cobalt(II) butane-2,3-diol monosolvate

**DOI:** 10.1107/S1600536811009147

**Published:** 2011-03-15

**Authors:** Shi-Juan Wang, Kai-Long Zhong

**Affiliations:** a Department of Applied Chemistry, Nanjing College of Chemical Technology, Nanjing 210048, People’s Republic of China

## Abstract

In the title compound, [Co(SO_4_)(C_12_H_8_N_2_)_2_]·C_4_H_10_O_2_, the Co^2+^ ion has a distorted octa­hedral coordination environment composed of four N atoms from two chelating 1,10-phenanthroline ligands and two O atoms from an *O*,*O*′-bidentate sulfate anion. The dihedral angle between the two chelating N_2_C_2_ groups is 83.48 (1)°. The Co^2+^ ion, the S atom and the mid-point of the central C—C bond of the butane-2,3-diol solvent mol­ecule are situated on twofold rotation axes. The mol­ecules of the complex and the solvent mol­ecules are held together by pairs of symmetry-related O—H⋯O hydrogen bonds with the uncoordinated O atoms of the sulfate ions as acceptors. The solvent mol­ecule is disordered over two sets of sites with site occupancies of 0.40 and 0.60.

## Related literature

For the ethane-1,2-diol solvate of the title complex, see: Zhong *et al.* (2006 ▶). For the propane-1,3-diol solvate of the title complex, see: Zhong (2010 ▶). For background to coordination polymers, see: Batten & Robson (1998 ▶); Lu *et al.* (2006 ▶); Zhang *et al.* (2010 ▶); Zhong *et al.* (2011 ▶).
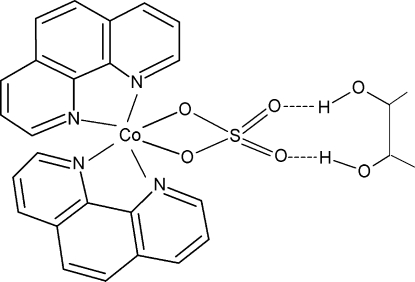


## Experimental

### 

#### Crystal data

[Co(SO_4_)(C_12_H_8_N_2_)_2_]·C_4_H_10_O_2_
*M* *_r_* = 605.52
Monoclinic, 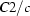

*a* = 18.184 (4) Å
*b* = 13.009 (3) Å
*c* = 13.112 (3) Å
β = 122.13 (3)°
*V* = 2626.6 (13) Å^3^
*Z* = 4
Mo *K*α radiation
μ = 0.79 mm^−1^
*T* = 223 K
0.40 × 0.20 × 0.10 mm

#### Data collection

Rigaku Mercury CCD diffractometer
Absorption correction: multi-scan (*REQAB*; Jacobson, 1998 ▶) *T*_min_ = 0.763, *T*_max_ = 1.000
7651 measured reflections
2316 independent reflections
2079 reflections with *I* > 2σ(*I*)
*R*_int_ = 0.033

#### Refinement

*R*[*F* ^2^ > 2σ(*F* ^2^)] = 0.046
*wR*(*F* ^2^) = 0.109
*S* = 1.12
2316 reflections
209 parameters
50 restraints
H-atom parameters constrained
Δρ_max_ = 0.49 e Å^−3^
Δρ_min_ = −0.39 e Å^−3^

### 

Data collection: *CrystalClear* (Rigaku, 2007 ▶); cell refinement: *CrystalClear*; data reduction: *CrystalClear*; program(s) used to solve structure: *SHELXS97* (Sheldrick, 2008 ▶); program(s) used to refine structure: *SHELXL97* (Sheldrick, 2008 ▶); molecular graphics: *XP* in *SHELXTL* (Sheldrick, 2008 ▶); software used to prepare material for publication: *SHELXTL*.

## Supplementary Material

Crystal structure: contains datablocks global, I. DOI: 10.1107/S1600536811009147/wm2463sup1.cif

Structure factors: contains datablocks I. DOI: 10.1107/S1600536811009147/wm2463Isup2.hkl

Additional supplementary materials:  crystallographic information; 3D view; checkCIF report

## Figures and Tables

**Table 1:** Selected bond lengths (Å)

Co1—O1	2.124 (2)
Co1—N1	2.128 (3)
Co1—N2	2.146 (2)
S1—O2	1.455 (2)
S1—O1	1.492 (2)

**Table 2:** Hydrogen-bond geometry (Å, °)

*D*—H⋯*A*	*D*—H	H⋯*A*	*D*⋯*A*	*D*—H⋯*A*
O3—H3⋯O2	0.82	2.07	2.779 (8)	145
O3′—H3′⋯O2	0.82	1.94	2.709 (7)	155

**Fig. 1.**
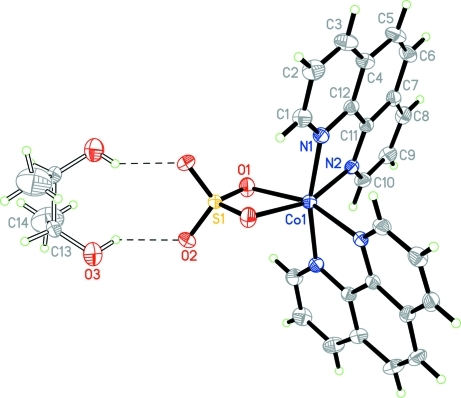
The molecular entities of the title compound, showing the atom-numbering scheme and with displacement ellipsoids drawn at the 35% probability level. The light broken lines depict O—H···O interactions. Unlabelled atoms are related to the labelled atoms by the symmetry operator (-x + 1, y, -z + 1/2). Note that only one orientation of the disordered solvent molecule is shown.

## supplementary crystallographic information

### Comment

The self-assembly of coordination polymers and the crystal engineering of metal-organic coordination frameworks have recently attracted great interest, owing to their interesting structural topologies and their potential applications as functional materials (Batten & Robson, 1998; Lu *et al.*, 2006; Zhang *et al.*, 2010; Zhong *et al.*, 2011). 1,10-phenanthroline (phen) has been widely used as an auxiliary ligands in constructing interesting coordination polymers. During attempts to synthesize mixed-ligand coordination polymers of transition metals with phen as second ligand *via* solvothermal reactions, we obtained compounds with a structure composed of a bidentate-chelating sulfate ligand, *e.g.* [CoSO_4_(C_12_H_8_N_2_)_2_].C_2_H_6_O_2_ (Zhong *et al.*, 2006), (II) and [CoSO_4_(C_12_H_8_N_2_)_2_].C_3_H_8_O_2_ (Zhong, 2010), (III).

We here report the new title compound, [CoSO_4_(C_12_H_8_N_2_)_2_].C_4_H_10_O_2_, (I), as part of our systematic investigation of transition metal complexes with bidentate bridging sulfate ligands. The crystal structure of the title compound consist of a neutral [CoSO_4_(C_10_H_8_N_2_)_2_] complex and a solvent butane-2,3-diol molecule. Twofold rotation axes pass through the Co and S atoms and the mid-point of the central C—C bond of the butane-2,3-diol solvent molecule. In the complex molecule, the Co^II^ ion is sixfold coordinated by four N atoms from two phen ligands and two O atoms from an *O,O*'-bidentate sulfate ion, giving rise to a distorted CoN_4_O_2_ octahedral environment (Fig. 1). The dihedral angle between the two chelating N_2_C_2_ groups is 83.48 (1)°, which is much larger that found in the structure of (II) and (III) (70.16 (6)° and 80.06 (8)°, respectively). The Co—O bond length [2.124 (2) Å], the O—Co—O bite angle [66.83 (12)°], the Co—N bond lenghts [2.128 (3)–2.146 (2) Å], and the N—Co—N bite angle [77.82 (10)°] are in good agreement with those observed in (II) [2.131 (1) Å, 66.32 (7)°, 2.126 (2)–2.137 (2) Å, 78.10 (6)°] and (III) [2.132 (2) Å, 66.54 (8)°, 2.123 (2)–2.134 (2) Å, and 77.99 (6)°].

The neutral metal complex and the solvent molecule are held together by pairs of symmetry-related O—H···O hydrogen bonds (Fig.1).

### Experimental

0.2 mmol 1,10-phenanthroline (phen), 0.1 mmol melamine, 0.1 mmol CoSO_4_.7H_2_O, 2.0 ml butane-2,3-diol and 1.0 ml water were mixed and placed in a thick Pyrex tube, which was sealed and heated to 423 K for 96 h. The tube was cooled to ambient temperature spontaneously and orange prism-shaped single crystals of the title compound were obtained.

### Refinement

All H atoms were positioned geometrically and allowed to ride on their attached atoms, with C—H(Ar) = 0.93 Å, C—H(CH) = 0.98 Å, C—H(CH_3_) = 0.96 Å and O—H = 0.82 Å; *U*_iso_(H) = 1.2*U*_eq_(C) and 1.5*U*_eq_(O). The solvent molecule butane-2,3-diol is disordered over two sets of sites and was refined with fixed site occupancies of 0.40 and 0.60.

### Crystal data

**Table:**

[Co(SO_4_)(C_12_H_8_N_2_)_2_]·C_4_H_10_O_2_	*F*(000) = 1252
*M**_r_* = 605.52	*D*_x_ = 1.531 Mg m^−^^3^
Monoclinic, *C*2/*c*	Mo *K*α radiation, λ = 0.71073 Å
Hall symbol: -C 2yc	Cell parameters from 4290 reflections
*a* = 18.184 (4) Å	θ = 3.1–27.5°
*b* = 13.009 (3) Å	µ = 0.79 mm^−^^1^
*c* = 13.112 (3) Å	*T* = 223 K
β = 122.13 (3)°	Prism, orange
*V* = 2626.6 (13) Å^3^	0.40 × 0.20 × 0.10 mm
*Z* = 4	

### Data collection

**Table:**

Rigaku Mercury CCD diffractometer	2316 independent reflections
Radiation source: fine-focus sealed tube	2079 reflections with *I* > 2σ(*I*)
Graphite monochromator	*R*_int_ = 0.033
Detector resolution: 28.5714 pixels mm^-1^	θ_max_ = 25.0°, θ_min_ = 3.2°
ω scans	*h* = −19→21
Absorption correction: multi-scan (*REQAB*; Jacobson, 1998)	*k* = −15→13
*T*_min_ = 0.763, *T*_max_ = 1.000	*l* = −15→14
7651 measured reflections	

### Refinement

**Table:**

Refinement on *F*^2^	Primary atom site location: structure-invariant direct methods
Least-squares matrix: full	Secondary atom site location: difference Fourier map
*R*[*F*^2^ > 2σ(*F*^2^)] = 0.046	Hydrogen site location: inferred from neighbouring sites
*wR*(*F*^2^) = 0.109	H-atom parameters constrained
*S* = 1.12	*w* = 1/[σ^2^(*F*_o_^2^) + (0.049*P*)^2^ + 4.2601*P*] where *P* = (*F*_o_^2^ + 2*F*_c_^2^)/3
2316 reflections	(Δ/σ)_max_ = 0.001
209 parameters	Δρ_max_ = 0.49 e Å^−^^3^
50 restraints	Δρ_min_ = −0.39 e Å^−^^3^

### Special details

**Table:**

Geometry. All e.s.d.'s (except the e.s.d. in the dihedral angle between two l.s. planes) are estimated using the full covariance matrix. The cell e.s.d.'s are taken into account individually in the estimation of e.s.d.'s in distances, angles and torsion angles; correlations between e.s.d.'s in cell parameters are only used when they are defined by crystal symmetry. An approximate (isotropic) treatment of cell e.s.d.'s is used for estimating e.s.d.'s involving l.s. planes.
Refinement. Refinement of *F*^2^ against ALL reflections. The weighted *R*-factor *wR* and goodness of fit *S* are based on *F*^2^, conventional *R*-factors *R* are based on *F*, with *F* set to zero for negative *F*^2^. The threshold expression of *F*^2^ > σ(*F*^2^) is used only for calculating *R*-factors(gt) *etc*. and is not relevant to the choice of reflections for refinement. *R*-factors based on *F*^2^ are statistically about twice as large as those based on *F*, and *R*- factors based on ALL data will be even larger.

### Fractional atomic coordinates and isotropic or equivalent isotropic displacement parameters (Å^2^)

**Table:**

	*x*	*y*	*z*	*U*_iso_*/*U*_eq_	Occ. (<1)
Co1	0.5000	0.67718 (4)	0.2500	0.0274 (2)	
S1	0.5000	0.46963 (8)	0.2500	0.0289 (3)	
O1	0.44423 (14)	0.54091 (16)	0.14832 (19)	0.0389 (6)	
N2	0.40247 (16)	0.78032 (18)	0.1216 (2)	0.0284 (6)	
C10	0.3905 (2)	0.8150 (2)	0.0182 (3)	0.0356 (7)	
H10A	0.4292	0.7942	−0.0037	0.043*	
O2	0.55262 (15)	0.40620 (18)	0.2215 (2)	0.0461 (6)	
C11	0.34456 (19)	0.8101 (2)	0.1512 (3)	0.0280 (7)	
C7	0.27533 (19)	0.8780 (2)	0.0798 (3)	0.0324 (7)	
C9	0.3233 (2)	0.8808 (3)	−0.0584 (3)	0.0401 (8)	
H9A	0.3173	0.9027	−0.1299	0.048*	
C8	0.2657 (2)	0.9131 (2)	−0.0276 (3)	0.0391 (8)	
H8A	0.2209	0.9578	−0.0775	0.047*	
C6	0.2174 (2)	0.9049 (3)	0.1186 (3)	0.0400 (8)	
H6A	0.1723	0.9506	0.0726	0.048*	
C12	0.35381 (18)	0.7670 (2)	0.2586 (3)	0.0288 (7)	
C4	0.2945 (2)	0.7931 (3)	0.2918 (3)	0.0348 (8)	
C5	0.2268 (2)	0.8654 (3)	0.2200 (3)	0.0422 (9)	
H5A	0.1889	0.8853	0.2436	0.051*	
N1	0.41924 (16)	0.69769 (19)	0.3205 (2)	0.0303 (6)	
C2	0.3687 (2)	0.6731 (3)	0.4543 (3)	0.0462 (9)	
H2A	0.3747	0.6391	0.5207	0.055*	
C1	0.4256 (2)	0.6524 (3)	0.4161 (3)	0.0385 (8)	
H1A	0.4698	0.6050	0.4592	0.046*	
C3	0.3041 (2)	0.7438 (3)	0.3930 (3)	0.0458 (9)	
H3A	0.2665	0.7592	0.4186	0.055*	
C13	0.5467 (3)	0.1136 (6)	0.2621 (9)	0.042 (2)	0.40
H13	0.5819	0.0562	0.3132	0.050*	0.40
O3	0.5935 (6)	0.2007 (6)	0.2842 (8)	0.066 (2)	0.40
H3	0.5605	0.2492	0.2500	0.098*	0.40
C14	0.4923 (11)	0.0849 (17)	0.1279 (9)	0.107 (6)	0.40
H14A	0.4601	0.0232	0.1180	0.161*	0.40
H14B	0.5300	0.0737	0.0986	0.161*	0.40
H14C	0.4527	0.1398	0.0834	0.161*	0.40
C13'	0.5031 (6)	0.1256 (6)	0.1915 (8)	0.081 (3)	0.60
H13'	0.4457	0.1395	0.1200	0.098*	0.60
O3'	0.5627 (6)	0.2002 (5)	0.2013 (8)	0.108 (3)	0.60
H3'	0.5454	0.2576	0.2046	0.161*	0.60
C14'	0.5388 (6)	0.0259 (6)	0.1741 (8)	0.073 (2)	0.60
H14D	0.5066	−0.0311	0.1771	0.110*	0.60
H14E	0.5989	0.0190	0.2368	0.110*	0.60
H14F	0.5336	0.0270	0.0972	0.110*	0.60

### Atomic displacement parameters (Å^2^)

**Table:**

	*U*^11^	*U*^22^	*U*^33^	*U*^12^	*U*^13^	*U*^23^
Co1	0.0229 (3)	0.0270 (3)	0.0300 (3)	0.000	0.0127 (3)	0.000
S1	0.0217 (5)	0.0260 (5)	0.0365 (6)	0.000	0.0137 (5)	0.000
O1	0.0324 (12)	0.0314 (12)	0.0327 (12)	−0.0003 (9)	0.0036 (10)	−0.0013 (9)
N2	0.0268 (13)	0.0261 (13)	0.0323 (14)	−0.0019 (10)	0.0156 (11)	0.0016 (10)
C10	0.0346 (17)	0.0382 (18)	0.0388 (18)	−0.0013 (14)	0.0228 (15)	0.0036 (14)
O2	0.0431 (13)	0.0377 (13)	0.0724 (17)	0.0066 (11)	0.0407 (13)	−0.0007 (12)
C11	0.0243 (15)	0.0262 (15)	0.0305 (15)	−0.0025 (12)	0.0125 (13)	−0.0010 (12)
C7	0.0262 (15)	0.0294 (16)	0.0320 (16)	0.0001 (13)	0.0090 (13)	−0.0022 (13)
C9	0.0383 (18)	0.045 (2)	0.0320 (17)	−0.0023 (15)	0.0150 (15)	0.0097 (15)
C8	0.0316 (17)	0.0344 (18)	0.0360 (18)	0.0010 (14)	0.0077 (15)	0.0082 (14)
C6	0.0263 (16)	0.0421 (19)	0.0406 (19)	0.0105 (14)	0.0103 (15)	0.0000 (15)
C12	0.0258 (15)	0.0287 (16)	0.0282 (15)	−0.0033 (12)	0.0119 (13)	−0.0027 (12)
C4	0.0297 (16)	0.0431 (19)	0.0307 (17)	0.0015 (14)	0.0155 (14)	−0.0028 (13)
C5	0.0289 (17)	0.056 (2)	0.0408 (19)	0.0083 (16)	0.0176 (15)	−0.0063 (16)
N1	0.0277 (13)	0.0300 (14)	0.0307 (13)	0.0016 (11)	0.0138 (11)	0.0022 (11)
C2	0.049 (2)	0.055 (2)	0.0374 (19)	−0.0005 (18)	0.0252 (17)	0.0071 (16)
C1	0.0388 (18)	0.0420 (19)	0.0346 (17)	0.0031 (15)	0.0195 (15)	0.0077 (14)
C3	0.0413 (19)	0.065 (2)	0.0401 (19)	0.0044 (18)	0.0276 (17)	0.0001 (18)
C13	0.035 (5)	0.028 (4)	0.060 (6)	0.004 (4)	0.024 (4)	0.000 (4)
O3	0.069 (5)	0.045 (4)	0.084 (5)	0.006 (3)	0.042 (4)	0.012 (4)
C14	0.116 (10)	0.148 (11)	0.090 (8)	−0.006 (8)	0.077 (8)	0.007 (8)
C13'	0.095 (6)	0.065 (5)	0.100 (7)	−0.003 (5)	0.062 (6)	0.015 (5)
O3'	0.148 (6)	0.052 (3)	0.178 (7)	−0.009 (4)	0.123 (6)	−0.015 (5)
C14'	0.089 (5)	0.068 (5)	0.081 (5)	−0.011 (4)	0.057 (4)	−0.007 (4)

### Geometric parameters (Å, °)

**Table:**

Co1—O1	2.124 (2)	C4—C3	1.399 (5)
Co1—O1^i^	2.124 (2)	C4—C5	1.434 (5)
Co1—N1	2.128 (3)	C5—H5A	0.9300
Co1—N1^i^	2.128 (3)	N1—C1	1.334 (4)
Co1—N2^i^	2.146 (2)	C2—C3	1.367 (5)
Co1—N2	2.146 (2)	C2—C1	1.392 (5)
S1—O2^i^	1.455 (2)	C2—H2A	0.9300
S1—O2	1.455 (2)	C1—H1A	0.9300
S1—O1^i^	1.492 (2)	C3—H3A	0.9300
S1—O1	1.492 (2)	C13—O3	1.353 (8)
N2—C10	1.332 (4)	C13—C14	1.537 (4)
N2—C11	1.359 (4)	C13—C13^i^	1.553 (5)
C10—C9	1.389 (5)	C13—H13	0.9800
C10—H10A	0.9300	O3—H3	0.8200
C11—C7	1.410 (4)	C14—H14A	0.9600
C11—C12	1.440 (4)	C14—H14B	0.9600
C7—C8	1.400 (5)	C14—H14C	0.9600
C7—C6	1.435 (5)	C13'—O3'	1.410 (4)
C9—C8	1.373 (5)	C13'—C14'	1.522 (8)
C9—H9A	0.9300	C13'—C13'^i^	1.596 (13)
C8—H8A	0.9300	C13'—H13'	0.9800
C6—C5	1.349 (5)	O3'—H3'	0.8200
C6—H6A	0.9300	C14'—H14D	0.9600
C12—N1	1.364 (4)	C14'—H14E	0.9600
C12—C4	1.402 (5)	C14'—H14F	0.9600
			
O1—Co1—O1^i^	66.83 (12)	C5—C6—C7	121.7 (3)
O1—Co1—N1	99.67 (10)	C5—C6—H6A	119.2
O1^i^—Co1—N1	92.38 (10)	C7—C6—H6A	119.2
O1—Co1—N1^i^	92.38 (10)	N1—C12—C4	123.2 (3)
O1^i^—Co1—N1^i^	99.67 (10)	N1—C12—C11	116.8 (3)
N1—Co1—N1^i^	165.59 (14)	C4—C12—C11	120.0 (3)
O1—Co1—N2^i^	159.07 (9)	C3—C4—C12	117.0 (3)
O1^i^—Co1—N2^i^	96.31 (9)	C3—C4—C5	123.6 (3)
N1—Co1—N2^i^	93.10 (10)	C12—C4—C5	119.4 (3)
N1^i^—Co1—N2^i^	77.82 (10)	C6—C5—C4	120.7 (3)
O1—Co1—N2	96.31 (9)	C6—C5—H5A	119.7
O1^i^—Co1—N2	159.07 (9)	C4—C5—H5A	119.7
N1—Co1—N2	77.82 (10)	C1—N1—C12	117.6 (3)
N1^i^—Co1—N2	93.10 (10)	C1—N1—Co1	128.2 (2)
N2^i^—Co1—N2	102.59 (13)	C12—N1—Co1	114.2 (2)
O1—Co1—S1	33.41 (6)	C3—C2—C1	119.3 (3)
O1^i^—Co1—S1	33.41 (6)	C3—C2—H2A	120.4
N1—Co1—S1	97.21 (7)	C1—C2—H2A	120.4
N1^i^—Co1—S1	97.21 (7)	N1—C1—C2	122.9 (3)
N2^i^—Co1—S1	128.71 (7)	N1—C1—H1A	118.6
N2—Co1—S1	128.71 (7)	C2—C1—H1A	118.6
O2^i^—S1—O2	110.9 (2)	C2—C3—C4	120.0 (3)
O2^i^—S1—O1^i^	110.50 (14)	C2—C3—H3A	120.0
O2—S1—O1^i^	110.77 (14)	C4—C3—H3A	120.0
O2^i^—S1—O1	110.77 (14)	O3—C13—C14	113.4 (11)
O2—S1—O1	110.50 (14)	O3—C13—C13^i^	122.6 (5)
O1^i^—S1—O1	103.17 (17)	C14—C13—C13^i^	78.3 (9)
O2^i^—S1—Co1	124.56 (10)	O3—C13—H13	112.7
O2—S1—Co1	124.56 (10)	C14—C13—H13	112.7
O1^i^—S1—Co1	51.59 (9)	C13^i^—C13—H13	112.7
O1—S1—Co1	51.59 (9)	C13—O3—H3	109.5
S1—O1—Co1	95.00 (11)	C13—C14—H14A	109.5
C10—N2—C11	117.6 (3)	C13—C14—H14B	109.5
C10—N2—Co1	129.0 (2)	H14A—C14—H14B	109.5
C11—N2—Co1	113.38 (19)	C13—C14—H14C	109.5
N2—C10—C9	123.3 (3)	H14A—C14—H14C	109.5
N2—C10—H10A	118.4	H14B—C14—H14C	109.5
C9—C10—H10A	118.4	O3'—C13'—C14'	103.3 (7)
N2—C11—C7	122.9 (3)	O3'—C13'—C13'^i^	110.9 (8)
N2—C11—C12	117.6 (3)	C14'—C13'—C13'^i^	113.5 (6)
C7—C11—C12	119.5 (3)	O3'—C13'—H13'	109.7
C8—C7—C11	117.5 (3)	C14'—C13'—H13'	109.7
C8—C7—C6	123.7 (3)	C13'^i^—C13'—H13'	109.7
C11—C7—C6	118.8 (3)	C13'—O3'—H3'	109.5
C8—C9—C10	119.4 (3)	C13'—C14'—H14D	109.5
C8—C9—H9A	120.3	C13'—C14'—H14E	109.5
C10—C9—H9A	120.3	H14D—C14'—H14E	109.5
C9—C8—C7	119.3 (3)	C13'—C14'—H14F	109.5
C9—C8—H8A	120.3	H14D—C14'—H14F	109.5
C7—C8—H8A	120.3	H14E—C14'—H14F	109.5
			
O1—Co1—S1—O2^i^	−90.19 (18)	C10—N2—C11—C12	175.8 (3)
O1^i^—Co1—S1—O2^i^	89.81 (18)	Co1—N2—C11—C12	−2.3 (3)
N1—Co1—S1—O2^i^	6.45 (14)	N2—C11—C7—C8	1.8 (4)
N1^i^—Co1—S1—O2^i^	−173.55 (14)	C12—C11—C7—C8	−176.2 (3)
N2^i^—Co1—S1—O2^i^	106.30 (16)	N2—C11—C7—C6	179.9 (3)
N2—Co1—S1—O2^i^	−73.70 (16)	C12—C11—C7—C6	2.0 (4)
O1—Co1—S1—O2	89.81 (18)	N2—C10—C9—C8	0.4 (5)
O1^i^—Co1—S1—O2	−90.19 (18)	C10—C9—C8—C7	−0.8 (5)
N1—Co1—S1—O2	−173.55 (14)	C11—C7—C8—C9	−0.2 (5)
N1^i^—Co1—S1—O2	6.45 (14)	C6—C7—C8—C9	−178.2 (3)
N2^i^—Co1—S1—O2	−73.70 (16)	C8—C7—C6—C5	176.8 (3)
N2—Co1—S1—O2	106.30 (16)	C11—C7—C6—C5	−1.2 (5)
O1—Co1—S1—O1^i^	180.0	N2—C11—C12—N1	−1.4 (4)
N1—Co1—S1—O1^i^	−83.36 (14)	C7—C11—C12—N1	176.7 (3)
N1^i^—Co1—S1—O1^i^	96.64 (14)	N2—C11—C12—C4	−178.4 (3)
N2^i^—Co1—S1—O1^i^	16.50 (15)	C7—C11—C12—C4	−0.4 (4)
N2—Co1—S1—O1^i^	−163.50 (15)	N1—C12—C4—C3	−0.6 (5)
O1^i^—Co1—S1—O1	180.0	C11—C12—C4—C3	176.3 (3)
N1—Co1—S1—O1	96.64 (14)	N1—C12—C4—C5	−178.8 (3)
N1^i^—Co1—S1—O1	−83.36 (14)	C11—C12—C4—C5	−2.0 (4)
N2^i^—Co1—S1—O1	−163.50 (15)	C7—C6—C5—C4	−1.2 (5)
N2—Co1—S1—O1	16.50 (15)	C3—C4—C5—C6	−175.3 (3)
O2^i^—S1—O1—Co1	118.26 (13)	C12—C4—C5—C6	2.8 (5)
O2—S1—O1—Co1	−118.45 (13)	C4—C12—N1—C1	0.8 (4)
O1^i^—S1—O1—Co1	0.0	C11—C12—N1—C1	−176.1 (3)
O1^i^—Co1—O1—S1	0.0	C4—C12—N1—Co1	−178.7 (2)
N1—Co1—O1—S1	−88.45 (12)	C11—C12—N1—Co1	4.4 (3)
N1^i^—Co1—O1—S1	99.50 (12)	O1—Co1—N1—C1	81.9 (3)
N2^i^—Co1—O1—S1	38.3 (3)	O1^i^—Co1—N1—C1	15.0 (3)
N2—Co1—O1—S1	−167.12 (12)	N1^i^—Co1—N1—C1	−131.8 (3)
O1—Co1—N2—C10	−75.8 (3)	N2^i^—Co1—N1—C1	−81.4 (3)
O1^i^—Co1—N2—C10	−110.8 (3)	N2—Co1—N1—C1	176.4 (3)
N1—Co1—N2—C10	−174.3 (3)	S1—Co1—N1—C1	48.2 (3)
N1^i^—Co1—N2—C10	17.0 (3)	O1—Co1—N1—C12	−98.7 (2)
N2^i^—Co1—N2—C10	95.2 (3)	O1^i^—Co1—N1—C12	−165.5 (2)
S1—Co1—N2—C10	−84.8 (3)	N1^i^—Co1—N1—C12	47.65 (19)
O1—Co1—N2—C11	102.0 (2)	N2^i^—Co1—N1—C12	98.0 (2)
O1^i^—Co1—N2—C11	67.0 (3)	N2—Co1—N1—C12	−4.2 (2)
N1—Co1—N2—C11	3.46 (19)	S1—Co1—N1—C12	−132.35 (19)
N1^i^—Co1—N2—C11	−165.2 (2)	C12—N1—C1—C2	0.0 (5)
N2^i^—Co1—N2—C11	−87.0 (2)	Co1—N1—C1—C2	179.4 (3)
S1—Co1—N2—C11	93.0 (2)	C3—C2—C1—N1	−1.0 (6)
C11—N2—C10—C9	1.1 (5)	C1—C2—C3—C4	1.3 (6)
Co1—N2—C10—C9	178.8 (2)	C12—C4—C3—C2	−0.5 (5)
C10—N2—C11—C7	−2.2 (4)	C5—C4—C3—C2	177.7 (3)
Co1—N2—C11—C7	179.7 (2)		

Symmetry codes: (i) −*x*+1, *y*, −*z*+1/2.

### Hydrogen-bond geometry (Å, °)

**Table:**

*D*—H···*A*	*D*—H	H···*A*	*D*···*A*	*D*—H···*A*
O3—H3···O2	0.82	2.07	2.779 (8)	145
O3'—H3'···O2	0.82	1.94	2.709 (7)	155
